# Stem Cell Therapy and Congenital Heart Disease

**DOI:** 10.3390/jcdd3030024

**Published:** 2016-07-05

**Authors:** Timothy J. Nelson, Susana Cantero Peral

**Affiliations:** 1 Division of General Internal Medicine, Mayo Clinic, Rochester, MN 55905, USA; canteroperal.susana@mayo.edu; 2 Department of Molecular Pharmacology and Experimental Therapeutics, Mayo Clinic, Rochester, MN 55905, USA; 3 Transplant Center, Mayo Clinic, Rochester, MN 55905, USA; 4 Center for Regenerative Medicine, Mayo Clinic, Rochester, MN 55905, USA

**Keywords:** congenital heart disease, regenerative medicine, stem cell therapy, heart failure

## Abstract

For more than a decade, stem cell therapy has been the focus of intensive efforts for the treatment of adult heart disease, and now has promise for treating the pediatric population. On the basis of encouraging results in the adult field, the application of stem cell-based strategies in children with congenital heart disease (CHD) opens a new therapy paradigm. To date, the safety and efficacy of stem cell-based products to promote cardiac repair and recovery in dilated cardiomyopathy and structural heart disease in infants have been primarily demonstrated in scattered clinical case reports, and supported by a few relevant pre-clinical models. Recently the TICAP trial has shown the safety and feasibility of intracoronary infusion of autologous cardiosphere-derived cells in children with hypoplastic left heart syndrome. A focus on preemptive cardiac regeneration in the pediatric setting may offer new insights as to the timing of surgery, location of cell-based delivery, and type of cell-based regeneration that could further inform acquired cardiac disease applications. Here, we review the current knowledge on the field of stem cell therapy and tissue engineering in children with CHD, and discuss the gaps and future perspectives on cell-based strategies to treat patients with CHD.

## 1. Introduction

Congenital heart disease (CHD) represents a significant burden on the family and community despite considerable advances in surgical techniques and clinical management. CHD occurs in ~7–8 out of 1000 live births [1]. There is still substantial morbidity and mortality related with CHD—mainly with the severe forms—which constitutes the leading cause of mortality due to congenital malformations [2]. In the last decade, the improvement in the management of CHD in advanced societies has been realized in more adults with CHD than children. Recent estimations reveal that up to 80% of newborns and infants with CHD are likely to reach adulthood [2], which can result in a high likelihood for complications later in life. Progressive late heart failure in children and young adults has become a serious problem, with an overall mortality of 7% in the United States [3]. The majority of these patients have CHD, with other causes being cardiomyopathy and myocarditis. In response to injury, the pediatric heart can undergo apoptosis, progressing to heart failure. Pressure overload in the children’s right ventricle results in a substantial increase in the production of cardiac stem cells, suggesting an adaptive response in this cohort of patients. However, this innate regeneration may not be sufficient to address the challenges of severe CHD [4]. Progressive heart failure can be accelerated in severe forms of CHD within the first few years of life after several challenges—despite supportive treatment—eventually resulting in the requirement for a heart transplant. Unfortunately, the supply of available hearts remains small compared to the number of patients in need. Therefore, to improve the clinical outcomes of these patients, advanced technologies are urgently required. Furthermore, there are new cell-based technologies that could be used for CHD applications [5]. There is an emerging realization that cardiac regeneration can occur in children, and that children are likely to respond to cell-based cardiac regenerative methods.

This review article addresses the current knowledge in the new field of stem cell-based regenerative therapies and tissue engineering and their potential to treat children with CHD when benchmarked to the experience in the adult practice, and describes the gaps in knowledge in the field of CHD repairment.

## 2. Stem Cells to Improve Cardiac Function in Adult Heart Disease

There is extensive experience with multiple types of cell-based products that are in development to mitigate the effects of acute and chronic ischemic heart diseases in adult populations. In the last two decades, attempts to treat adult heart failure with stem cells—mainly involving the administration of autologous bone marrow derived stem cells (BMSC)—have demonstrated a consistent safety profile without evidence of increased arrhythmogenicity or tumor formation. These results are highlighted in several published meta-analyses [6,7,8,9,10,11,12,13,14]. Stem cell therapy has shown promising results in adults with ischemic heart disease. However, these results have been inconsistent across a clinical spectrum of acquired heart disease. Besides the inconsistency in the results, various studies have shown sustained positive effects despite consistent evidence that infused or injected cells do not survive beyond 30 days in vivo. The use of stem cell therapy in adult heart disease has paved the road for the application of cell-based regenerative medicine in the pediatric setting.

Herein, we focus on the literature that reported on adults with heart disease that were treated using BMSC, to compare with current pediatric reports.

Strauer et al. [15] first reported intracoronary (IC) delivery of bone marrow-derived mononuclear cells (BM-MNCs) in acute ischemic patients in 2002. Following that trial, other clinical trials using BM-MNCs—including the TOPCARE-AMI trial [16,17], the BOOST trial [18,19,20], TCT-STAMI [21], REPAIR-AMI [22], ASTAMI [23,24], FINCELL [25], BALANCE [26], SCAMI [27], BONAMI [28], COMPARE-AMI [29], LateTIME [30], TIME [31], CARDIAC [32], IACT [33], TOPCARE-CHD [34], and STAR-heart [35]—have been conducted in acute and chronic ischemic heart disease settings. In Table 1 we show the feasibility, safety, and efficacy of these studies. These numerous clinical studies have demonstrated the safety of IC delivery of stem cells. Thousands of patients have been included in similar clinical trials and received the cells via IC infusion—the most common method of cell delivery in the clinical setting—which allows placement of the cells into myocardial regions. BMSC are the most broadly used stem cells in regenerative medicine since their discovery in the 1960s. They were first used in hematopoietic stem cell transplantation to replace diseased bone marrow. Bone marrow contains different types of hematopoietic and non-hematopoietic stem cells, resulting in a very useful source for its regenerative potential. Autologous BMSC are among the best described multipotent stem cells for transplantation because their use does not require immunosuppressive therapy and they are easily accessible. All the clinical studies reviewed herein involved autologous BMSC, with the majority being mononuclear cells. The main outcome is a high degree of confidence in the safety profile. These results are confirmed with numerous published meta-analyses (Table 2) [9,10,13,14,15,36]. Those studies concluded that there were no differences in major adverse events between bone marrow cell-treated and control groups, such as all-cause mortality, cardiac mortality, incidence of recurrent myocardial infarction, and in-stent thrombosis, a potential concern in patients treated with IC BMSC infusion. The incidence of other important clinical adverse outcomes—including target vessel revascularization and ventricular arrhythmia—also did not differ between groups. Some of the meta-analyses reviewed found that the number of adverse events were actually significantly lower in the cell therapy group [9,10,13].

In a recently published meta-analysis [8], the safety and efficacy of IC cell therapy after acute myocardial infarction (AMI) have been analyzed, including individual patient data (*n* = 1252) from 12 randomized clinical trials. Except for one study, all patients received BM-MNCs. As found in other meta-analyses published before, there was no effect of cell therapy on major adverse cardiac and cerebrovascular events, or death. However, regarding efficacy, this first prospectively declared collaborative multinational database has revealed that IC cell therapy provided no clinical benefit or changes in left ventricular function. Another meta-analysis reported by de Jong et al. [6]—where 2037 patients were included from 30 randomized controlled trials—proved cell therapy also to be safe. BM-MNC therapy showed a slight improvement in left ventricular ejection fraction (LVEF), mainly because of a sustained left ventricular end-systolic volume (LVESV), along with a reduced infarct size. However, when those studies were analyzed using cardiac magnetic resonance imaging-based measurements, no functional improvements in cardiac function, volume, or infarct size were demonstrated. In previous meta-analyses, IC infusion of BM-MNCs resulted in a mild-moderate cardiac function after acute ischemic disease. When newer large randomized controlled clinical trials restricted to patients with AMI were included in the latest meta-analyses, the IC infusion of BM-MNCs did not show an improvement of cardiac function or a reduction of all-cause mortality, cardiac mortality, or recurrent AMI hospitalizations for either cardiac or neurologic complications.

These efforts and the contradictory results have led to a large European multicenter, randomized open-label, controlled, parallel-group phase III trial (BAMI study-NCT01569178). The aim of this definitive study for adult patients is to demonstrate that a single intracoronary infusion of autologous BM-MNCs is safe and reduces all-cause mortality in adults with reduced LVEF (≤45%) after successful reperfusion for AMI when compared to a control group of patients undergoing the best medical care. This study plans to enroll 3000 patients.

From a total of 45 thoroughly-reviewed clinical trials with more than 2000 patients included—the majority being randomized controlled trials—we herein highlight three of these studies based on the existing heart disease of the patients enrolled in the studies, cell-based products, and delivery strategies relevant to the documented pediatric experience [37,38,39,40] (Table 3). The patients included in these three clinical studies had non-ischemic heart diseases; in two cases they suffered from non-ischemic idiopathic dilated cardiomyopathy (DCM), and in one case from chronic chagasic cardiomyopathy. In two of those studies, BM-MNCs (unfractionated bone marrow) were used as the cell therapy, equivalent to the majority of pediatric clinical cases reported in the literature. Similar to reported results in previous clinical trials with ischemic patients, there was no increase in mortality or severe adverse events in the bone marrow cell-treated group. In the ABCD study [38,39], at three-year follow-up there was significant sustained improvement of LVEF. The Miheart-Chagas study [37] did not demonstrate any improvement in LVEF in the stem cell therapy group, with similar mortality in both groups. The main outcome showed by Vrtovec et al. was that in the bone marrow cell group, the total mortality was lower when compared with the control group after 60 months of follow-up (14% vs. 31%, *p* = 0.01). Overall, the reported safety results have been similar between non-ischemic heart patients and ischemic heart populations.

## 3. Cell-Based Therapy Experience in Patients with CHD or Heart Failure

On the basis of the experience and early results in adult patients, as we mentioned above, the application of stem cells to patients with CHD creates the opportunity to explore a new therapeutic paradigm.

Experience with stem cell therapy in children with severe congenital or acquired heart failure is not extensive (Table 4). To date, no large clinical trials have been published using stem cells to repair CHD. Most of the reports published used BMSC via IC infusions. To date, the two pediatric populations targeted to receive cardiac cell therapy have been patients with DCM and patients with single ventricle congenital heart defects. The first case reports using stem cell therapy in children with CHD were based on the extensive experience in adults with reduced left ventricle systolic function. However, the main causes of children’s heart damage and heart failure are very different from those that cause heart failure in the adult population, who typically have multiple comorbidities. Pediatric heart diseases are more commonly secondary to CHD, infections, inflammation, cytotoxicity, or immune disorders. It is important to emphasize that pediatric patients can present with dilated and poorly functioning hearts, yet recover dramatically over a period of months with supportive care when the etiology is myocarditis, as opposed to adult patients. In the cases discussed in this review, none of the patients treated with cell therapy had myocarditis. Therefore, parameters such as age of individual, stem cell delivery strategy, and disease status, could lead to distinctive performance features of cell-based technologies.

The first case of cell-based therapy in a child with DCM was reported by Rupp et al. in 2009 [41]. The authors reported that IC injection of BM-MNCs was safe and feasible. The LVEF improved from 24% to 45% after 6 months of cell injection. One year later, the same group published a new case report of cell therapy in an 11-month-old patient. There were no complications during the cell infusion and no adverse events were reported. At 3-months follow-up, the cardiac function had improved, showing a reduction of end-diastolic and end-systolic volumes [42]. Most recently, Rupp et al. [43] reported the results of IC BM-MNCs delivery for terminal heart failure in nine children to stabilize the end-stage heart failure condition of all patients as compassionate use. Two-thirds of the patients suffered from DCM and one-third was affected with CHD. Moderate ST-segment changes were reported during stem cell delivery. However, no increase in cardiac enzymes or unexpected adverse events were observed after the procedure. After donor organs became available, two patients proceeded to heart transplantation (at 48 and 32 days after cell therapy), before the efficacy of the procedure could be demonstrated. Three patients with DCM and two patients with CHD benefited from autologous infusion of BMSC. They showed an improvement in their clinical condition, and in LVEF.

In 2010, Olguntürk et al. [44] published a report of two pediatric patients with DCM who received peripheral blood-derived mononuclear cells after granulocyte-colony stimulating factor (G-CSF) via the coronary arteries. Both cases were referred to the clinic for cardiac transplantation due to end-stage heart failure that was resistant to drugs. The cells were collected by leukopheresis after the patients received G-CSF (10 ug/kg/day) for four days, until the CD34+ cell count reached 30 × 10^9^/L. Total mononuclear cells infused were 1.96 × 10^6^/kg and 1.27 × 10^6^/kg, respectively. In patient one, auto-limited ventricular tachycardia was observed during the procedure, probably related with rapid infusion. No other adverse events were observed in either patient. Five weeks after the cell infusion, the patients’ clinical status improved considerably in parallel to the echocardiographic results. At eight weeks, patient one went from 16% of LVEF (at time of admission) to 39%, and patient two from 34% to 51%. At six months, patient two showed an LVEF of 54%, and this patient was removed from the heart transplantation list with New York Heart Association (NYHA) class I status.

In 2011, Lacis et al. [45] reported—for the first time—the intramyocardial administration of autologous BM-MNCs in a four-month-old infant with severe DCM. At four-month follow-up, the LVEF had increased from 20% (before stem cell transplantation) to 41%.

In 2013, Bergmane et al. [46] published the first pediatric cohort with a one-year follow-up of six patients out of seven that were diagnosed with DCM and received BMSC. Seventeen to 122 × 10^6^ BMSC were isolated. There were no side effects upon cell delivery. The average basal LVEF was 33.5%, and an increase of up to 54% was observed at 6-month and 12-month follow-up.

In 2010, the first pediatric case of transcoronary injection of bone marrow-derived progenitor cells for end-stage heart disease after a myocardial infarction was reported. The nine-year-old patient received bone marrow CD133+/CD34+ cells using a transcoronary catheter without any complication. Three months after the cell therapy treatment, the LVEF (by cardiac magnetic resonance and echocardiogram) improved from 30% at baseline to 47% [47].

Altogether, these first studies offer an encouraging perspective on the potential for first-generation stem cell therapy to be considered in the pediatric population as an adjunctive therapy to surgical management of CHD.

In March of 2013, our group launched the first USA-based stem cell trial for CHD (NCT01883076), with the main goal being the determination of the safety and feasibility of autologous umbilical cord blood (UCB)-derived stem cells for cardiac regeneration in children with HLHS. We have reported the first case of direct intramyocardial injection of umbilical cord blood-derived mononuclear cells (UCB-MNCs) in an infant with HLHS [48]. The UCB was collected at the time of delivery, and the MNCs fraction was isolated and stored in liquid nitrogen. The cells were injected into the right ventricle at the time of the Glenn procedure. No adverse events occurred either at the time of infusion or later. Transthoracic echocardiography at three months showed improvement in right ventricular systolic function, with an estimated ejection fraction of 50%, increased from 30%–35% before surgery. Since 2015, our group has been conducting a phase I study (NCT02549625) to determine the safety and feasibility of intracoronary delivery of autologous BM-MNCs in individuals, from 2–30 years of age, with Fontan circulation and declining ventricle systemic pumps.

Since April 2013, a randomized-controlled, prospective phase II clinical trial has been conducted at Okayama University in Japan (PERSEUS-NCT01829750). The Cardiac Progenitor Cell Infusion to Treat Univentricular Heart Disease (PERSEUS) trial has been designed to assess the efficacy of intracoronary infusion of cardiac progenitor cells (CDCs) in young patients (up to 20 years of age) with univentricular heart disease (HLHS, single right ventricle and single left ventricle). A total of 34 patients are randomly assigned 1:1 to the treated or control group. Patients included in this study are in a preoperative high-risk group or did not recover cardiac function postoperatively, therefore, eventually their only option is heart transplantation. This phase II clinical study has been implemented following the safety verification of the previous phase I study (TICAP trial) completed in January 2013 by the same investigators, and published recently by Tarui et al. [49]. In this controlled study, 14 consecutive patients with HLHS were prospectively assigned to receive intracoronary CDCs one month after cardiac surgery (*n* = 7), followed by seven control patients who received standard care alone. The cell infusion was feasible and no serious adverse events were reported within 36 months of follow-up. Echocardiography showed a significant right ventricular ejection fraction (RVEF) improvement in those infants who received CDCs compared with the controls. Other ongoing clinical trials using stem cells in CHD include phase I trials at Duke University (NCT01445041) and at University of Miami (NCT02398604). The Duke study, which has temporarily suspended participant recruitment, is evaluating the safety and feasibility of collecting and infusing intravenously autologous UCB in newborns with HLHS. In addition, as a secondary goal, the investigators will evaluate the efficacy of the UCB-MNCs to improve the neurological function affected in these children. Recently, the University of Miami has begun enrollment of HLHS pediatric patients in a phase I trial to deliver intramyocardially allogeneic mesenchymal stem cells (MSCs) during the bi-directional cavopulmonary anastomosis surgery. Thirty patients are intended to be enrolled. The first 10 patients will receive allogeneic MSCs to determine feasibility and safety. The next 20 HLHS patients will be randomized to the treatment and control arms in a 1:3 ratio, respectively.

## 4. Translational Cell-Based Research for Cardiac Repair in CHD 

As the reported adult experience and pre-clinical studies [50,51,52,53] have shown us, cell-based regenerative therapies have been manufactured from a broad range of cell sources including bone marrow, cord blood, peripheral blood, and CDC. Beyond the first-generation of cell-based products, there is a growing list of cells, growth factors, and genetic engineering strategies that have been pioneered in the adult cardiac practice of regenerative medicine [54]. Cell-based products have been delivered either intra-myocardium or intra-coronary showing a safety range with no evidence of adverse events resultant to cell type or delivery strategy. Pediatric response to stem cells has resulted in measurable improvements in cardiac function, albeit in a limited number of patients to-date. It is almost impossible not to compare and contrast the adult and pediatric experiences in order to measure the magnitude of benefit that cell-based therapy could have for CHD. However, the main concern about this promising technology is the limited data due to the isolated cases reported. The clinical trials emerging will offer an equable protocol design and the ability to collect experimental evidence to advance research and clinical development utilizing regenerative strategies for CHD.

The potential of cell therapy in pediatric patients with CHD is enormous with different challenges not observed in adults. Due to the differences in pathobiology between the pediatric and adult cardiac diseases, it is difficult to anticipate the efficacy of stem cell therapy in CHD based on adult strategies, and thus it requires empiric testing in pre-clinical settings. With the new established large animal model systems we are able to carry out double-blinded, randomized studies to test safety and efficacy in the pediatric stages of disease.

Regarding the delivery strategy, the pediatric population with CHD requires multiple open-chest surgeries that allow for direct intramyocardial injection of stem cell-based products. Therefore, the delivery strategy in the diseased pediatric heart will be different than the usual intracoronary infusion in adults with ischemic heart disease, providing a focal and direct access to the myocardium.

As we described above, several types of stem cells are being used for cardiac regenerative medicine, and most of the clinical trials used in adult population have been conducted with autologous cells. This approach has the main advantage of avoiding immunologic reaction. In the pediatric population this is even more important, because a large number of these patients with CHD or acquired DCM could eventually require a heart transplant when the traditional and new therapeutic strategies are not effective. The chances to find a compatible donor if the patient has been sensitized with allogeneic antigens could be dramatically reduced. Furthermore, animal and clinical studies have demonstrated that aging interferes with progenitor cell functions and potency [55]. Stem cells from young individuals possess superior naivety and plasticity than stem cells from adults. Apoptosis and DNA damage increase in aging stem cells, and those defects can further reduce the pool of undifferentiated and progenitor cells. Edelberg et al. first reported that age directly affects cell-mediated improvement of new blood vessels, and demonstrated that young—not old—bone marrow cells were incorporated into the new vasculature and restored angiogenic cardiac functions [56]. Aging is also linked to a reduction of telomere length. The use of autologous cells in the pediatric setting—due the age of these patients—seems to be an advantage compared with older cells and better suited for regenerative purposes.

Fruitful regenerative strategies for pediatric patients with CHD should result in significant de novo cardiogenesis and remuscularization of the abated heart tissue. Uncertainty persists about the possible mechanisms by which stem cells might enhance cardiac function. Initially, it was believed that stem cells promoted cardiac differentiation by tissue replacement due to stem cell direct differentiation into cardiomyocytes [57]. More recently, several studies have revealed that first-generation cell-based product transplantation in heart disease stimulates an endogenous cardiac repair by releasing cytokines and growth factors following a paracrine effect [58,59]. Therefore, those cell products need to be prioritized in the pediatric setting. A paracrine effect added to the beneficial results of stem cell therapy supports the hypothesis that the combination of cytokines, chemokines and growth factors with cell therapy may have a synergistic effect on cardiac repair. Therapeutic interventions using just chemokines and growth factors are now the focus of subsequent investigations [60]. Based on this, these first-generation cell-based products may be sufficient to launch clinical services and provide clinically relevant results. Furthermore, second-generation products that include reprogrammed progenitor cells and/or combinations with biomaterials, still require testing in the pediatric population. The use of stem cells with biomaterials in the CHD setting may provide additional features in the context of reconstructive procedures. Finally, bioengineered pluripotent stem cells have proved to be the only type of stem cells able to remuscularize the heart muscle with de novo tissues. As high-risk structural heart defects in children mandate new functioning tissue, induced pluripotent stem cell-based strategies will likely provide the most meaningful strategy for a long-term functional cure and offer a unique opportunity to execute the first induced pluripotent stem cell-based clinical trial in a “no-option” population. Engineering strategies are needed to demonstrate the safety profile of pluripotent stem cells, along with pre-clinical testing in small and large animals prior to clinical studies.

CHD has now entered into the field of stem cell-based regenerative medicine. The emerging cell-based products and biomaterials have synergistic function, requiring safety and efficacy preclinical studies with the goal of moving towards innovative clinical trials. The need to apply the right cells, at the right time, to the right patient, will prioritize the experimental questions and experimental designs in the coming years, offering a new horizon for deterrent regenerative cardiac therapies.

## 5. Conclusions

In the field of CHD, several types of stem cells have been used with promising results. However, stem cell therapy strategies for the pediatric population with heart failure has just begun; therefore, further clinical trial studies will be needed to understand the cell biology in order to optimize their regenerative potential. The key to ideal cardiac regenerative cell therapy would be to combine different strategies, such as priming stem cells, combined with chemokines, and bio-engineering materials. Therefore, multiple cell-based products and strategies need to be evaluated head-to-head in specific pre-clinical models with clinically relevant delivery strategies to identify the optimal manufactured product for the right person at the right time. The challenge for the field of CHD regeneration is to build sustainable synergy between clinical practice and discovery science to prioritize the people, processes, and technology with a singular focus. This unmet clinical demand will need collaboration between academia, biobusiness companies, and governmental agencies to take advantage of the resources and expertise in order to safely translate research discoveries into clinical solutions and accelerate the next generation cell-based technologies for CHD.

## Figures and Tables

**Table 1:** Literature review of intracoronary delivery of autologous bone marrow stem cells in adult patients with ischemic heart disease.

Study	Patients/Control	Study Design	Cell Type Infused	Days from Disease to Cells Infusion	Follow-up (Months)	Efficacy Outcomes
Strauer et al., 2002 [15]	10/10	C	MNCs	5–9 days	3	No significant LVEF improvement vs. control. Significant improvement with regard to infarct region, hemodynamics, cardiac geometry, and contractility.
**TOPCARE-AMI trial**						
Assmus et al., 2002 [16]	9(BM)/11(PB)	R-NC	BM-MNCs & PB-MNCs	4.3 ± 1.5 days	4	LVEF: cell therapy group > non-randomized matched reference group.
						No difference in LVEF between BM and PB groups.
Schachinger et al., 2004 [17]	29(BM)/30(PB)	R-NC	BM-MNCs & PB-MNCs	4.9 ± 1.5 days	12	Cell therapy was associated with significant improvements in LVEF, and significant reductions in LV end-systolic volumes after one year of myocardial infarction.
**BOOST trial**						
Wollert et al., 2004 [20]	30/30	RC	MNCs	4.8 ± 1.3 days	6	Improvement in LVEF in bone marrow group.
Meyer et al., 2006 [18]	30/30	RC	MNCs	4.8 ± 1.3 days	18	BM group showed improvement in LVEF at 6 months, not sustainable after 18 months.
Meyer et al., 2009 [19]	30/30	RC	MNCs	4.8 ± 1.3 days	60 (28/28 patients)	There is an early improvement of diastolic function without a sustained effect on long-term follow-up.
**TCT-STAMI**						
Ge et al., 2006[21]	10/10	R-CDB	MNCs	1 day	6	BM cells after AMI improved cardiac function.
**REPAIR-AMI trial**						
Assmuss et al., 2010 [22]	101/103	R-PCDB	MNCs	4 ± 1 days	24	Infusion of BM cells improved LV contractile function and protected against heart failure in the 2 years after stem cell therapy.
**ASTAMI**						
Lunde et al., 2008 [24]	50/50	R-PC	MNCs	6 days	6, 12, 36	At 3 years, it was just found a small improvement in exercise time in the BM group, with no other remarkably signs of improvement.
Beitnes et al., 2011 [23]						
**FINCELL**						
Huikuri et al., 2008 [25]	40/40	R-PC	MNCs	2–6 days	6	At 6 months, LVEF increased in the BM group compared with the placebo group.
**BALANCE**						
Yousef et al., 2009 [26]	62/62	C	MNCs	7 ± 2 days	3, 12, 60	At 3-months follow-up, BM group showed a significant improvement of LVEF and stroke volume index. The infarct size was significantly reduced by 8%. Those parameters were stable at 12 and 60 months. The mortality was significantly reduced in the BM cell therapy group compared with the control group.
**SCAMI**						
Wohrle et al., 2013 [27]	29/13	R-PCDB	MNCs	5–7 days	1, 3, 6, 36	Improvement in LVEF up to 3 years in patients who received high doses of BM cells or without microvascular obstruction.
**BONAMI**						
Roncalli et al., 2011 [28]	52/49	RC	MNCs	9.3 ± 1.7 days	3	Improvement of myocardial viability in multivariate analysis.
**COMPARE-AMI**						
Mansour et al., 2011 [29]	20/20	R-CDB	MNCs-CD133+	6.4 ± 2.2 days	12	LVEF significantly improved at four months of follow up and remained higher at 12 months.
**LateTIME**						
Traverse et al., 2011 [30]	58/29	R-PCDB	MNCs	14–21 days	6	No improvement in regional function or LVEF.
**TIME**						
Traverse et al., 2012 [31]	3 days: 43/24	R-PCDB	MNCs	3 vs. 7	6	No differences on LVEF between BM and placebo groups.
	7 days: 36/17					
**CARDIAC**						
Piepoli et al., 2013 [32]	19/19	RC	CD45+ & MNCs	4 days	3, 6, 12, 24	Significant improvement in LVEF at 12 month follow-up in the BM group, not found at 24 months.
**IACT**						
*Strauer* et al., 2005 [33]	18/18	C	MNCs	3 months to 9 years	3	Improvement in LVEF and reduced infarct size by 30% in the BM group.
**TOPCARE-CHD**						
Assmus et al., 2006 [34]	24/28/23/PB/BM/Control	RCC	PB-MNCs & BM-MNCs	>90 days (2470 ± 2196 days)	3	Significant improvement in LVEF in the BM group at 3-month follow-up. No improvement in the PB group when compared with placebo.
**STAR-heart**						
Strauer et al., 2010 [35]	191/200	C	MNCs	8.5 ± 3.2 years	3, 12, 60	At 5-year follow-up, improvement in LVEF and increased survival in the BM group.

**Table 2:** Summary of meta-analysis studies for intracoronary stem cell transplantation in acute ischemic heart disease.

Authors/Year	Disease	Number of Studies Included	Study Design	Total # of Patients Included	Cell Type	Follow-up Duration	Major Adverse Events in Stem Cell Group Compared with Controls
Gyongyosi et al., 2015 [8]	AMI	12	RCT	1252	BM-MNCs (*n* = 11)	Mean: 3–12 months	No (1)
					Cardiosphere-derived cells (*n* = 1)		
de Jong R et al., 2014 [6]	AMI	30	RCT	2037 (1218 cell therapy vs. 819 controls)	BM-MNCs (*n* = 22)	Median: 6 months	No (2)
					MSCs (*n* = 3)		
					BM CD133+ CD34+ (*n* = 4)		
					Cardiosphere-derived cells (*n* = 1)		
Delewi et al., 2014 [7]	AMI	16	RCT	1641 (984 cell therapy vs. 657 controls)	BM-MNCs (*n* = 13)	3–6 months	No (3)
					BM-CD34+/CXCR4+ (*n* = 1)		
					Nucleated BM cells (*n* = 2)		
Jeevanantham et al., 2012 [9]	IHD (AMI & CIHD)	50 (38 IC vs. 12 IM)	RCT (*n* = 36)	2625	BM-MNCs (*n* = 36)	3–60 months	No (4)
					BM-CD34+ and or CD133+ (*n* = 6)		
			CS (*n* = 14)		Nucleated BM cells (*n* = 5)		
					BM-MSC and/or endothelial progenitor cells (*n* = 3)		
Zimmet et al., 2012 [14]	AMI	29 (23 IC vs. 6 G-CSF trials)	RCT	1830 (1470 from IC trials)	BM stem cells	Short-term (3–6 months)	No (5)
						Long-term (12–18 months)	
Ye et al., 2012 [12]	AMI	10	RCT	757 (394 cell therapy vs. 363 controls)	BM-MNCs	Mean: 1–5 years	No (6)
Zhang et al., 2009 [13]	AMI	8	RCT	525	BM stem cells	1–5 years	No (7)
Martin-Rendon et al., 2008 [11]	AMI	13	RCT	811	BM-MNCs	3–6 months	No
Lipinski et al., 2007 [10]	AMI	10	Controlled trials	698	BM stem cells (*n* = 8)	3–18 months	No (8)
					PB mononuclear cells (*n* = 2)		

AMI: acute myocardial infarction; IHD: ischemic heart disease; CIHD: chronic ischemic heart disease; IC: intracoronary; IM: intramyocardial; BM: bone marrow; RCT: randomized controlled trials; CS: cohort studies; BM-MNCs: bone marrow mononuclear cells; BM-MSCs: bone marrow mesenchymal stem cells; PB: peripheral blood; MI: myocardial infarction; LVEF: left ventricular ejection fraction. (1) This meta-analysis of individual patient data revealed that IC cell therapy provided no benefit, in terms of clinical events or changes in LVF; (2) IC infusion of BM-MNCs is safe, but does not enhance cardiac function of MRI-derived parameters, nor does it improve clinical outcome; (3) IC BMC therapy leads to a modest but significant improvement of LVEF. Patients of younger age and with a more severely depressed LVEF showed the largest benefit; (4) BM cells transplantation reduced the incidence of death, recurrent MI, and stent thrombosis; (5) Lower revascularization rates with IC BM stem cells vs. control; (6) Sustained and moderate improvements of LVEF and attenuations of infarct size; (7) BM cell group significantly reduced the risk of death; (8) BM cell group showed a trend to reduce major adverse events.

**Table 3:** Randomized controlled studies with bone marrow mononuclear cells for intracoronary delivery in adults with non-ischemic cardiomyopathies.

Study	Patients/Controls	Disease	Study Design	Cell Type and Dosage	Time from Disease to BM Infusion	Follow-up (Months)	Outcome
**ABCD trial**							
Seth et al., 2010 [38]	45/40	Non-ischemic idiopathic DCM	RC	MNCs 1.68 × 10^8^	>6 months	36	LVEF improved in the BM group by 5.9% from 6-month follow-up with a reduction in end-systolic volumes and no change in end-diastolic volumes.
**Miheart-Chagas**							
Ribeiro dos Santos et al., 2012 [37]	117/117	Chronic chagasic cardiomyopathy	R-PC	MNCs 2.2 × 10^8^	Not available	6, 12	No improvement in LVEF
							Mortality was similar in both groups
Vrtovec et al., 2013 [40]	55/55	Non-ischemic DCM	RC	MNCs CD34+ 113 ± 26 × 10^6^	>3 months	60	Intracoronary BM stem cell infusion was associated with improved LVEF, exercise tolerance, and long-term survival at 5-year follow-up, and lower total mortality, when compared with control group.

BM: Bone marrow; MNCs: mononuclear cells; LVEF: left ventricular ejection fraction; DCM: dilated cardiomyopathy; RC: randomized controlled; R-PC: randomized placebo-controlled.

**Table 4:** Case reports and clinical trials of stem cell-based therapy in children with CHD and/or heart failure.

Study/Author	No of Patients	Age of Patients	Entity Cardiac Status	Study Design	Cell Type and Cell Dose	Delivery Route	Follow-up	Outcomes
Rupp S et al., 2009 [41]	1	2 years	DCM	Case report	Autologous BM-MNCs/20 × 10^6^ cells/kg	IC	6 months	Safe and feasible
								↑ LVEF
								↓ NYHA
								↓ BNP
Rupp S et al., 2010 [42]	1	11 months	HLHS + mitral stenosis + aortic atresia	Case report	Autologous BM-MNCs	IC	3 months	Safe and feasible
								↑ LVEF
								↓ BNP
Rupp S et al., 2012 [43]	9	4 months–16 years	DCM (*n* = 6) and CHD (*n* = 3)	Cohort	Autologous BM-MNCs	IC	24–52 months	1 pt = death no procedure-related
								3 pts = heart Tx
								5 pts =
								↑ LVEF
								↓ NYHA
								↓ BNP
Olguntürk et al., 2010 [44]	2	6 years, 9 years	DCM	Case reports	Autologous PB-MNCs mobilized with G-CSF/1.96 and 1.27 × 10^6^ cells/kg	IC	2–6 months	↑ LVEF
								↓ NYHA
								↓ BNP
								1 pt was removed of the heart Tx list
Lacis A et al., 2011 [45]	1	4 months	DCM	Case report	Autologous BM-MNCs	IM	4 months	↑ LVEF
Bergmane I et al., 2013 [46]	7 (6 completed follow-up)	4 months–17 years	DCM	Cohort	Autologous BM-MNCs	IC	12 months	Safe and feasible
								↑ LVEF
								↓ LVEDV
Limsuwan A et al., 2010 [47]	1	9 years	CHF after MI	Case report	Autologous BM-CD133+/CD34+ mobilized with G-CSF	IC	3 months	↑ LVEF ↓ NYHA
Burkhart H et al., 2014 [48]	1	4 months	HLHS	Case report	Autologous UCB-MNCs/3 × 10^6^ cells/kg	IM	3 months	↓ NYHA
								↑ RVEF
								↓ BNP
TICAP study, Okayama University, Japan	14 (7 cell therapy vs. 7 controls)	≤6 years 1.8 ± 1.5 years	HLHS	Phase 1 Prospective, controlled	Autologous CDC/0.3 × 10^6^ cells/kg	IC	36 months	Safe and feasible
								↑ RVEF
								↓ BNP
PERSEUS trial, Okayama University, Japan	34	≤20 years	Univentricular heart disease	Phase 2 Prospective, randomized-controlled	Autologous CDC/0.3 × 10^6^ cells/kg	IC	12 months	Ongoing, but not recruiting patients
								NCT01829750
Mayo Clinic, USA	10	≤18 months	HLHS	Phase 1	Autologous UCB-MNCs/3 × 10^6^ cells/kg	IM	6 months	Recruiting patients since 2013
								NCT01883076
Duke University, USA	20	≤2 days	HLHS	Phase 1, randomized	Autologous UCB cells 5 × 10^7^ TNC cells/kg	IV	12 months Focus in neurologic effects	Ongoing, but not recruiting patients
								NCT01445041
University of Miami, USA	30	≤28 days	HLHS	Phase 1, randomized after first 10 patients	Allogeneic MSCs/2.5 × 10^5^ cells/kg	IM	12 months	Recruiting patients since 2015
								NCT02398604
Mayo Clinic, USA	10	2–30 years	Single RV failure due to CHD	Phase 1	Autologous BM-MNCs/3 × 10^6^ cells/kg	IC	24 months	Recruiting patients since 2015
								NCT02549625

DCM: Dilated cardiomyopathy; BM-MNCs: Bone marrow-derived mononuclear cells; IC: intracoronary; LVEF: left ventricular ejection fraction; NYHA: New York Heart Association; BNP: Brain natriuretic peptide; HLHS: Hypoplastic left heart syndrome; CHD: congenital heart disease; pt: patient; pts: patients; Tx: transplant; PB-MNCs: Peripheral blood-derived mononuclear cells; G-CSF: Granulocyte-colony stimulating factor; LVEDV: left ventricular end-diastolic volume; IM: intramyocardial; RVEF: Right ventricular ejection fraction; CDC: cardiosphere-derived cells; TNC: Total nucleated cells; RV: right ventricle; MI: myocardial infarction.
